# Antimicrobial effects of zero-valent iron nanoparticles on gram-positive *Bacillus* strains and gram-negative *Escherichia coli* strains

**DOI:** 10.1186/s12951-017-0314-1

**Published:** 2017-11-03

**Authors:** Yi-Huang Hsueh, Ping-Han Tsai, Kuen-Song Lin, Wan-Ju Ke, Chao-Lung Chiang

**Affiliations:** 1 Graduate School of Biotechnology and Bioengineering, Yuan Ze University, Taoyuan, Taiwan; 2 Department of Chemical Engineering and Materials Science, Yuan Ze University, Taoyuan, Taiwan; 3 Department of Microbiology and Immunology, Chang Gung University, Taoyuan, Taiwan

**Keywords:** Zero-valent iron, Nanoparticles, Antimicrobial, *Bacillus*, *Escherichia coli*, Redox

## Abstract

**Background:**

Zero-valent iron nanoparticles (ZVI NPs) have been used extensively for the remediation of contaminated soil and groundwater. Owing to their large active surface area, they serve as strong and effective reductants. However, the ecotoxicity and bioavailability of ZVI NPs in diverse ecological media have not been evaluated in detail and most studies have focused on non-nano ZVI or Fe^0^. In addition, the antimicrobial properties of ZVI NPs have rarely been investigated, and the underlying mechanism of their toxicity remains unknown.

**Results:**

In the present study, we demonstrate that ZVI NPs exhibited significant toxicity at 1000 ppm against two distinct gram-positive bacterial strains (*Bacillus subtilis* 3610 and *Bacillus thuringiensis* 407) but not against two gram-negative strains (*Escherichia coli* K12 and ATCC11634). Specifically, ZVI NPs caused at least a 4-log and 1-log reductions in cell numbers, respectively, in the two *Bacillus* strains, whereas no change was detected in the two *E. coli* strains. X-ray photoelectron spectroscopy, X-ray absorption near-edge, and extended X-ray absorption fine structure spectra confirmed that *Bacillus* cells exposed to ZVI NPs contained mostly Fe_2_O_3_ with some detectable FeS. This finding indicated that Fe^0^ nanoparticles penetrated the bacterial cells, where they were subsequently oxidized to Fe_2_O_3_ and FeS. RedoxSensor analysis and propidium iodide (PI) staining showed decreased reductase activity and increased PI in both *Bacillus* strains treated with a high (1000 ppm) concentration of ZVI NPs.

**Conclusion:**

Taken together, these data show that the toxicity of ZVI NPs was derived from their oxidative properties, which may increase the levels of reactive oxygen species and lead to cell death.

## Background

Zero-valent iron nanoparticles (ZVI NPs) have been extensively used to remediate contaminated soil and groundwater because of their ability to target chlorinated organic compounds (e.g., polychlorinated biphenyl, trichloroethylene, perchloroethylene, pesticides, solvents), inorganic anions (e.g., perchlorate), and heavy metals [1–3]. ZVI NPs have a large active surface area, can serve as strong and effective reductants, and show an elevated capacity to absorb abundant heavy metals and contaminants [4]. There are many types of ZVI NPs, including uncapped, surface-modified, and bimetallic ZVI NPs, which can be used for the dechlorination of chlorinated solvents, in dense non-aqueous phase liquid source zones, and for odor control in biosolid treatments [5–8]. Other metal nanoparticles, such as silver, gold, and copper nanoparticles [9–11] have been reported to negatively affect bacterial growth and to kill bacteria in the soil [12, 13], through mechanisms that have been only partially characterized. At present, the toxicity of ZVI NPs towards bacteria and the underlying mechanism remain unclear.

The ecotoxicity and bioavailability of ZVI NPs in diverse ecological media have not been evaluated in detail, and the studies conducted to date have mostly focused on non-nano ZVI or Fe^0^. Although ZVI NPs themselves are nontoxic, they can oxidize Fe^0^ to Fe^2+^ and then to Fe^3+^, and these chemical forms are readily found in the habitats of soil microorganisms. In addition, application of ZVI NPs under oxidative conditions can considerably increase the local concentrations of Fe^2+^ and Fe^3+^. Consequently, oxidation of ZVI NPs leads to the production of reactive oxygen species (ROS), resulting in the generation of hydroxyl radicals (OH^−^) from superoxide (O_2_^−^) and hydrogen peroxide (H_2_O_2_) in microbial cells. These radicals enhance oxidative stress and cause cell membrane damage, leading to the outflow of intracellular contents and, ultimately, cell death [9, 14].

To date, there have been few reports on the toxicity of ZVI NPs. Some studies have shown the ability of ZVI NPs to inhibit the growth of *Escherichia coli* by disrupting its cell membrane [15–17], whereas other studies have shown that ZVI NPs smaller than 50 nm were not effective against the soil gram-negative bacterium *Pseudomonas stutzeri*, even when treated at 10,000 ppm for 48 h [18]. Diao and Yao [9] found that 20–30-nm ZVI NPs caused a 100% reduction (1000–10,000 ppm) in *Pseudomonas fluorescens* and 80–100% reduction (100–10,000 ppm) in *Bacillus subtilis* but had no effect (100–10,000 ppm) on the growth of *Aspergillus versicolor*.

There have been very few reports on the influence of ZVI NPs on complex microbial ecosystems [19–24]. It is therefore difficult to predict the effects of NPs on an ecological system, given that their behavior and environmental fate are poorly understood [13]. Considering the high daily fluctuations in environmental soil parameters, this represents an additional challenge to realistically simulate the complex soil environment in an experimental exposure scenario. Indeed, the mobility and toxicity of metal particles to soil microorganisms can be influenced by several natural soil parameters, such as pH, calcium concentration, organic matter content, C-to-N ratio, and cation exchange capacity [25, 26]. However, ZVI NPs can also have an inhibitory effect on biological dechlorination in the presence of 1 g/L iron nanoparticles, resulting in toxicity to indigenous bacteria that hinders their participation in the remediation process [9, 27]. Xiu et al. [27] found that ZVI NPs initially inhibited the growth of bacteria dechlorinating trichloroethylene, but their dechlorination activity and ethane production recovered after a lag period [27]. Given that ZVI NPs are highly redox-active metals [28], their environmental impact and safety when applied to the soil should be investigated in more detail [12, 29].

In the present study, we examined the antimicrobial activity of different concentrations of ZVI NPs against two common soil gram-positive bacteria, *Bacillus subtilis* 3610 and *B. thuringiensis* 407, as well as two common soil gram-negative bacteria, *Escherichia coli* K12 and ATCC11634, grown in Mueller Hinton broth (MHB). The potential mechanisms underlying toxicity were examined by X-ray absorption spectroscopy, which is an excellent tool to determine valence and local structure.

## Methods

### ZVI NP preparation

Ten grams of FeSO_4·_7H_2_O powder was dissolved in 70 mL deionized water with stirring at 220–260 rpm and then mixed with 30 mL ethanol. Next, 2.0 M NaOH aqueous solution was added dropwise until a pH value of 6.8 ± 0.1 was achieved. Next, 1.8 g of NaBH_4_ was dissolved in 5.0 mL deionized water and dropped in the solution with mild stirring for 30 min. A magnet was applied to separate the nano-iron particles thus generated from the solution. The filtered ZVI NPs were washed using deionized water and ethanol three times each. Finally, ZVI NPs were dried at room temperature using flowing N_2_ for 12 h.

### X-ray absorption near-edge structure (XANES) and extended X-ray absorption fine structure (EXAFS) analyses

Overnight cultures containing approximately 1 × 10^9^ colony-forming units (CFU)/mL of *E. coli* K12, *E. coli* ATCC11634, *B. subtilis* 3610, and *B. thuringiensis* 407 were diluted 100-fold into 250 mL of MHB in four 500-mL Pyrex flasks. ZVI NPs were added to a concentration of 1000 ppm. Cells were grown for 3 h at 37 °C and 175 rpm in a rotary shaker, after which they were centrifuged at 5000 rpm for 15 min, the supernatant was discarded, and cells were washed with double-distilled H_2_O. Finally, samples were freeze-dried into powder. XANES/EXAFS spectra were collected on the Wiggler beam line 17C1 at the National Synchrotron Radiation and Research Center (NSRRC) of Taiwan. The electron storage ring was operated at an energy of 1.5 GeV and a ring current of 100–200 mA. Data were collected in transmission mode by a Lytle detector in the region of the Fe *K*-edge at 25 °C. Data were subsequently normalized using Athena (vi) software, with the linear pre-edge and polynomial post-edge backgrounds subtracted from the raw ln(*I*_*t*_/*I*_0_) data (where *I*_*t*_ is the light intensity after it passes through the sample and *I*_0_ is the initial light intensity), and then analyzed using Artemis (vi) software with FFEF-8. The spectra were first energy-calibrated by simultaneously recording the transmission spectra of the Fe foil with Athena (vi), where the energy of the first inflection point for the reference sample absorption edge was 7112 eV. After calibration, samples were background-corrected (using a linear pre-edge region and a polynomial for the post-edge region) and normalized. EXAFS energy spectra were then converted into wavevector *K*-space form. Data directly reflected the average local environment around the absorbing atoms. Spectra were analyzed using the IFEFFIT [30, 31] software package. The theoretical paths for the Fe–Fe and Fe–O species were generated using the FEFF-8 program based on the crystallographic data of the individual species and were used to fit the first coordination shell of the experimental data. The coordination number, interatomic distance, Debye–Waller factor, and inner potential corrections were used as variables for the fitting procedures.

### ZVI NP characterization

The X-ray diffraction (XRD) patterns of the synthesized ZVI NP samples were recorded at a scan rate of 4° (2*θ*)/min using monochromatic Cu K_α_ radiation (MXP18; MAC Science, Japan) at 30 kV and 20 mA. The recorded specific peak intensity and 2*θ* values were further identified using a database system (JCPDS). The morphology, microstructure, and particle size of the ZVI NPs were investigated by field-emission scanning electron microscopy (FE-SEM; S-4700 Type II; Hitachi, Japan) and high-resolution transmission electron microscopy (HR-TEM; H-7500; Hitachi, Japan).

### Assessment of the antibacterial effects of ZVI NPs, Fe_2_O_3_, and oxidized Fe NPs

*Escherichia coli* K12, *E. coli* ATCC11634, *B. subtilis* 3610, and *B. thuringiensis* 407 strains were maintained in MHB on 1.5% Bacto agar plates at 37 °C. Overnight cultures of approximately 5 × 10^8^ CFU/mL were added in 50 mL MHB in 250-mL Pyrex flasks at pH 7. ZVI NPs were added to final concentrations of 0, 100, 500, and 1000 ppm. Cells were grown for 3 h at 37 °C and 175 rpm in a rotary shaker. All cultures were serially diluted, plated on Luria–Bertani (LB) agar plates, incubated overnight at 37 °C, and then subjected to a colony count. All experiments were performed in duplicate, and each value represents the mean of three technical replicates. Statistical significance was determined using one-way analysis of variance (ANOVA).

### RedoxSensor measurements

Reductase activities of *B. subtilis* 3610 and *B. thuringiensis* 407 were determined using a BacLight RedoxSensor Green Vitality Kit (ThermoFisher, Waltham, MA, USA). Overnight cultures of approximately 1 × 10^7^ CFU/mL in 50 mL MHB were supplemented with the indicated concentrations of ZVI NPs for 3 h at 175 rpm and 37 °C. Cells were washed with 1 × phosphate-buffered saline (PBS) twice, diluted 10-fold with the same buffer, and eventually mixed well with 1 μL of RedoxSensor Green reagent. Additionally, 1 μL of propidium iodide (PI) was added to the mixture, which was incubated in the dark at room temperature for 5 min before assessing cell membrane integrity. Stained cells (1 mL) in PBS were assayed with a FACSCalibur Flow Cytometer (BD Biosciences, San Jose, CA, USA). In addition, fluorescence filters and detectors were all standardized with green fluorescence collected in the FL1 channel (530 ± 15 nm) and red fluorescence collected in the FL3 channel (> 650 nm). Data were analyzed using FACSCalibur Flow Cytometer software. All parameters were collected as logarithmic signals. All measurements were performed in two separate experiments.

### X-ray photoelectron spectroscopy (XPS) analyses

The chemical composition on the surface of the as-synthesized ZVI NPs was determined by XPS (Physical Electronic ESCA PHI 1600; Chanhassen, MN, USA) at the excitation energy of Al K_α_ (1486.6 eV). The C 1s (284.5 eV) peak was used as the calibration standard for the wide-region spectra of these samples with different Fe valences [e.g. Fe(0), Fe(II), Fe(III)]. XPS signals of the above species were recorded with a cylindrical mirror analyzer (CMA). The fractions of these samples with different Fe valences were calculated by their integrated peak areas. All measurements were performed in two separate experiments.

## Results

### ZVI NPs inhibited growth

**Fig. 1:**
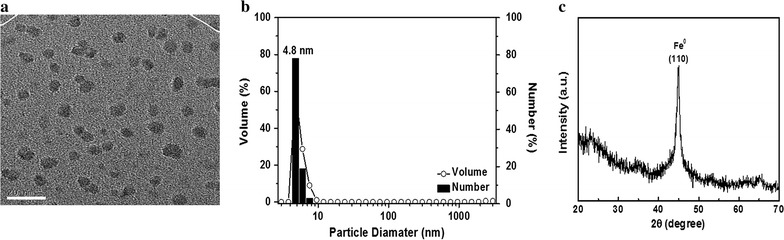
Shape, crystal structure, and particle size characterization of ZVI NPs. **a** SEM images of the ZVI NPs used in this study; white bar: 20 nm. **b** Size distribution histograms of ZVI NPs. **c** XRD patterns of the synthesized ZVI NPs. Data are representative of two separate experiments

ZVI NPs vary broadly in terms of size and morphology [32–34]. Thus, to compare the present results with those of previous studies, we first characterized the size and structure of the ZVI NPs synthesized using FE-SEM. As shown in Fig. 1a, b, ZVI NPs were about 4.8 nm in diameter on average. XRD patterns showed a main characteristic diffraction peak at 2θ = 44.7° (Fig. 1c) based on the diffraction patterns for Fe^0^ nanoparticles (JCPDS Card Number 06-0696, [35]). The overall XRD pattern was comparable with previously reported spectra.

**Fig. 2:**
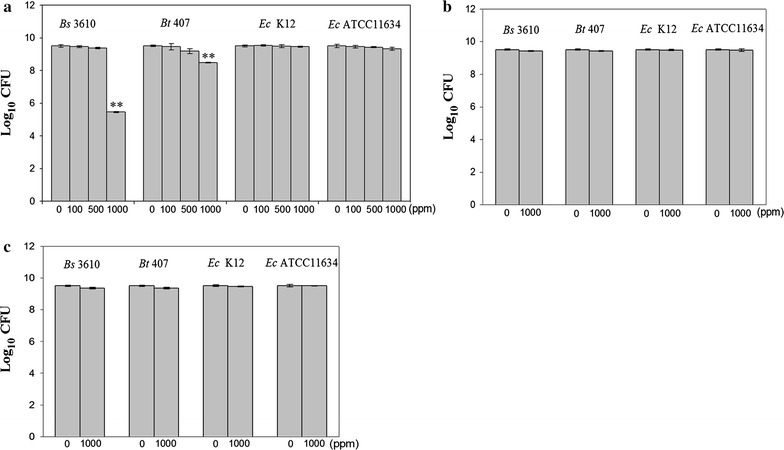
Antibacterial effects of different ZVI NP concentrations against *E. coli* and *Bacillus* strains. **a** *B. subtilis* 3610 (*Bs *3610), *B. thuringiensis* 407 (*Bt *407), *E. coli* K12 (*Ec* K12), and *E. coli* ATCC11634 (*Ec* ATCC11634) grown in MHB medium were treated with different concentrations of ZVI NPs at 37 °C with shaking at 175 rpm for 3 h; antibacterial activity was evaluated by plating and counting cells. *B. subtilis* 3610, *B. thuringiensis* 407, *E. coli* K12, and *E. coli* ATCC11634 grown in MHB medium were treated with different concentrations of **b** Fe_2_O_3_ **c** Oxidized Fe(ZVI) NPs at 37 °C with shaking at 175 rpm for 3 h; antibacterial activity was evaluated as above. Data are expressed as means ± standard deviations of two separate experiments, with three replicates. **p < 0.01 as determined by ANOVA relative to the control sample at different pH values

Next, we examined the antimicrobial activity of ZVI NPs against two gram-positive soil bacteria (*B. subtilis* 3610 and *B. thuringiensis* 407) and two gram-negative soil bacteria (*E. coli* K12 and ATCC11634). ZVI NPs did not significantly retard the growth of either *E. coli* strain, but inhibited both *Bacillus* strains (Fig. 2a). Specifically, *B. subtilis* 3610 exhibited a 4-log reduction, and *B. thuringiensis* 407 showed a 1-log reduction in the presence of 1000 ppm ZVI NPs (Fig. 2a), indicating that the latter was relatively more resistant. Collectively, these data suggested that ZVI NPs were more toxic to gram-positive than gram-negative bacteria. In addition, we next aimed to differentiate whether ZVI NP oxide entered cells and caused ROS generation, leading to cell death, or whether oxide ZVI NPs, such as Fe_2_O_3_, caused cell death. Therefore, ZVI NPs were shaken in MHB medium under aerobic conditions for 3 h, and we then examined oxidation forms. XPS and XANES analyses showed that most ZVI NPs became Fe_2_O_3_. We further used oxide ZVI NPs and Fe_2_O_3_ in antimicrobial assays and found that even 1000 ppm oxide ZVI NPs and Fe_2_O_3_ were unable to inhibit cell growth or death for all four strains (Fig. 2b, c). This suggested that ZVI NPs killed bacterial cells when the ZVI NPs entered cells and caused oxidation and ROS generation, thereby leading to cell death.

### ZVI NPs altered the redox status in *B. subtilis* and *B. thuringiensis*

**Fig. 3:**
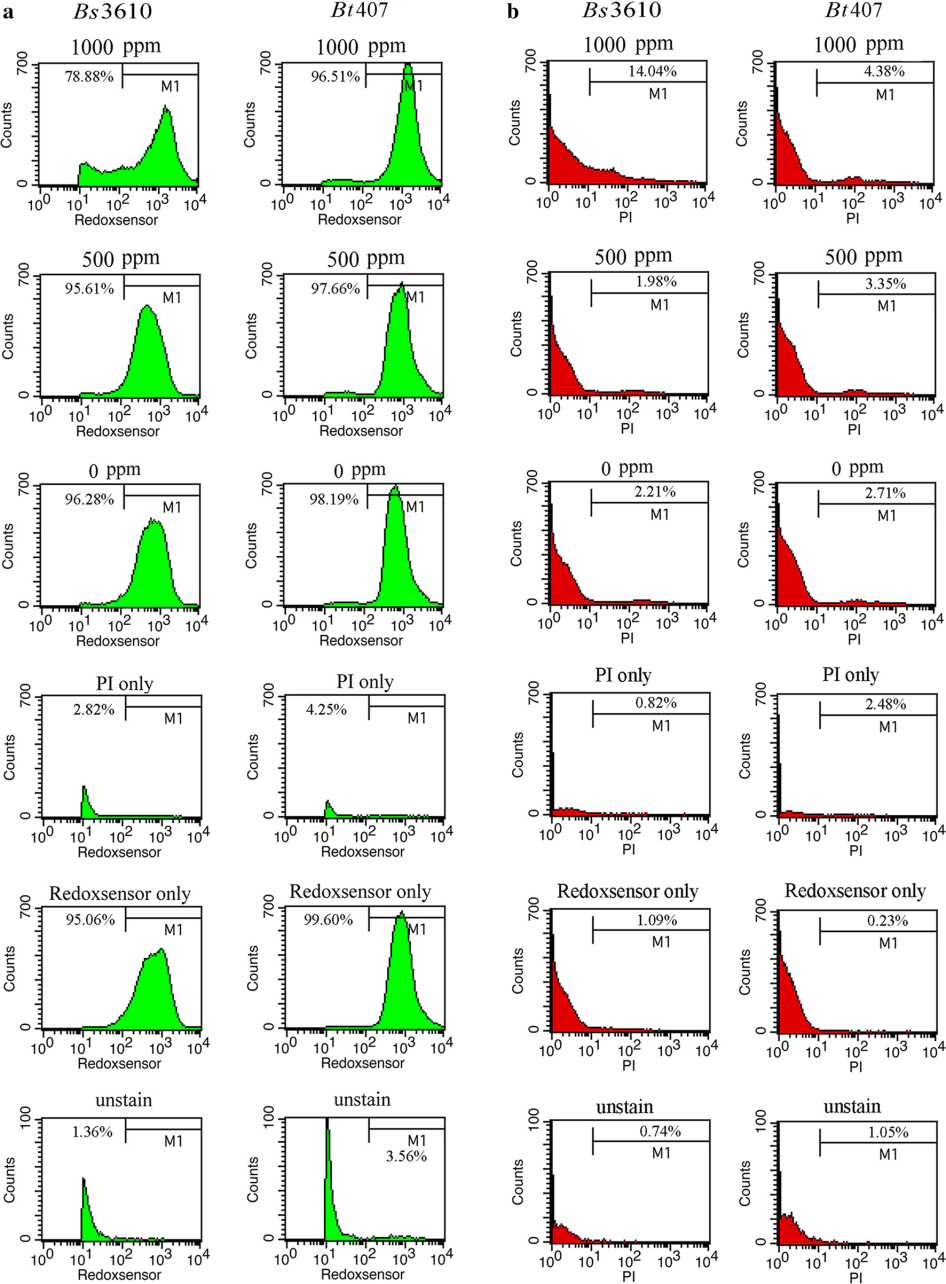
Analysis of RedoxSensor and propidium iodide (PI) staining intensity in *B. subtilis* 3610 (*Bs*3610) and *B. thuringiensis* 407 (*Bt*407). The two *Bacillus* strains were grown in MHB supplemented with ZVI NPs at concentrations of 0, 100, 500, and 1000 ppm for 3 h, and the cells were then treated with **a** RedoxSensor Green (green) or **b** PI (red). The X-axis shows the RedoxSensor or PI fluorescence intensity in arbitrary units (au), and the Y-axis indicates the cell counts as measured by flow cytometry. PBS-only and unstained cells were used as controls. Data are representative of two separate experiments. RedoxSensor fluorescence is presented in a false green color, and PI staining is presented in a false red color for clearer visualization

ZVI NPs may cause an increase in ROS; therefore, to examine the potential mechanisms of the observed growth inhibition [28], we evaluated whether these nanoparticles could affect reductase activity in the two *Bacillus* strains. Briefly, overnight bacterial cell cultures (~ 1 × 10^7^ CFU/mL) were treated with 0–1000 ppm ZVI NPs for 3 h and then stained with RedoxSensor Green, consisting of a green fluorescent dye to assess oxidoreductase activity and a red dye to assess membrane integrity. Accordingly, ZVI NPs reduced the percentage of cells (M1 Gate %) showing reductase activity in a concentration-dependent manner (Table 1, Fig. 3a) and dramatically increased the percentage of PI-positive cells (M1 Gate %; Fig. 3b). Thus, nanoparticles severely compromised the integrity of the cell membranes after 3 h, suggesting that, compared with the control, many cells were dead at 1000 ppm (Table 1, Fig. 3b).

**Table 1:** Flow cytometry analysis of *B. subtilis* 3610 and *B. thuringiensis* 407

Strain	Concentration	PI M1 Gate % = (PI count − background)/(total count) (%)	RedoxSensor M1 Gate % = (RedoxSensor count − background)/(total count) (%)	PI Geo mean (au)	RedoxSensor Geo mean (au)
*Bs*3610	Unstained	0.74	1.36	22.05	328.80
	1000 ppm	14.04	78.88	40.21	1002.53
	500 ppm	1.98	95.61	65.98	488.07
	0 ppm	2.21	96.28	94.18	594.69
	PI only	0.82	2.82	32.41	429.63
	RedoxSensor only	1.09	95.06	26.47	577.59
*B*t407	Unstained	1.05	3.56	23.09	580.79
	1000 ppm	4.38	96.51	93.19	1244.31
	500 ppm	3.35	97.66	90.06	783.37
	0 ppm	2.71	98.19	100.89	655.33
	PI only	2.48	4.25	54.80	412.67
	RedoxSensor only	0.23	99.60	23.03	767.53

### Oxidation states and fine structural parameters of ZVI NPs in *B. subtilis* 3610 and *B. thuringiensis* 407

To resolve whether the observed toxicity was derived from the ZVI NPs themselves or the consequent iron oxidation and production of intracellular ROS, we analyzed the Fe oxidation states of ZVI in the two *Bacillus* strains and two *E. coli* strains using XPS [36]. The findings are presented in Table 2 as the integrated area percentages of Fe(0), Fe(II), and Fe(III). The iron oxidation states on the surfaces of these samples were analyzed using XPS spectra, as illustrated in Table 2. In particular, we found that the ZVI NPs were oxidized to Fe(III) (60.8%) or Fe(II) (39.2%) after 3-h oxidation treatment in MHB medium. Since the ZVI NPs came in contact with dissolved oxygen from the atmosphere, it would be oxidized to iron oxides, such as FeO, Fe_2_O_3_, and Fe_3_O_4_. Thus, this phenomenon implied the high reduction ability of ZVI NPs in MHB medium for killing the bacteria. The iron species in the *B. subtilis* 3610 and *B. thuringiensis* 407 were identified as Fe(0) (17.6 and 11.9%), Fe(II) (27.0 and 26.9%), and Fe(III) (55.4 and 61.2%), respectively. The existence of Fe(0) was attributed to rapid absorption or diffusion of ZVI NPs into *B. subtilis* 3610 and *B. thuringiensis* 407, reducing the oxidization of ZVI NPs. In contrast, ZVI NPs in solution could not easily be absorbed or diffused through the outer bilayer cell membranes of *E. coli* K12 and *E. coli* ATCC11634, thus suppressing cytotoxicity.

**Table 2:** XPS analysis of *B. subtilis* 3610, *B. thuringiensis *407, oxidized Fe NPs, *E. coli* K12, and *E. coli* ATCC11634

Sample	Integrated area percentages of iron species (%)		
Fe species	Fe (2*p*_*3/2*_)		
	Fe(0)	Fe(II)	Fe(III)
*Bs* 3610	17.6	27.0	55.4
*Bt* 407	11.9	26.9	61.2
Oxidized Fe(ZVI) NPs	0.0	39.2	60.8
*Ec* K12	ND	ND	ND
*Ec* ATCC11634	ND	ND	ND

The Fe_2_O_3_ content was over 50% in both *Bacillus* strains, but the percentage of FeS was substantially lower. In addition, the Fe content was minimal in *Bacillus* strains

*ND* not detected

**Fig. 4:**
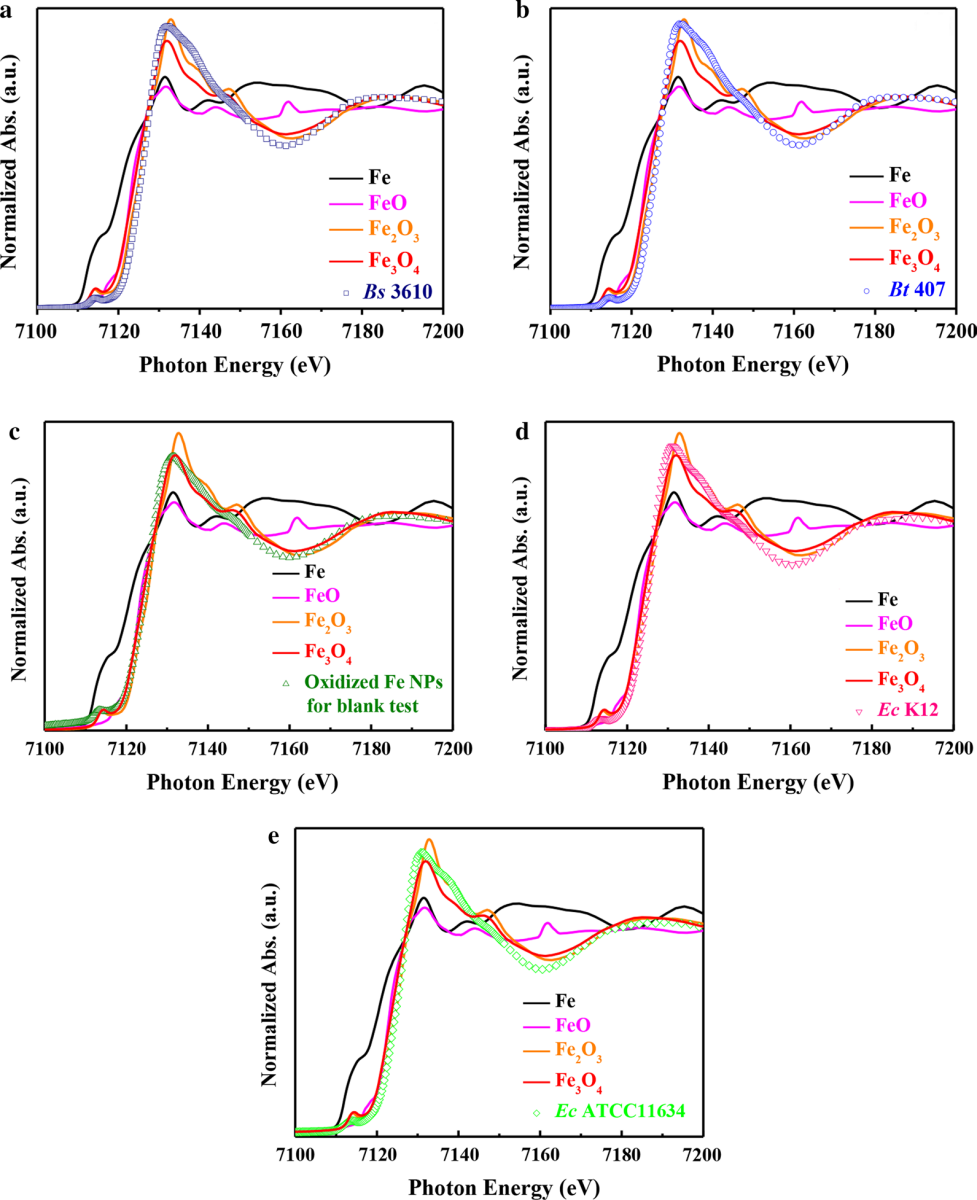
Normalized *K*-edge XANES spectra of ZVI NPs standards, *Bacillus* cells, and *E. coli* cells treated with 1000 ppm ZVI NPs. *K*-edge-normalized XANES spectra of **a** *B. subtilis* 3610 (*Bs *3610), **b** *B. thuringiensis* 407 (*Bt *407), **c** oxidized Fe(ZVI) NPs, **d** *E. coli* K12 (*Ec * K12), and **e** *E. coli* ATCC11634 (*Ec* ATCC11634). Data are representative of two separate experiments

Furthermore, the iron oxidation states in the internals of all *Bacillus* and *E. coli* cells were investigated with XANES spectra [37] in Fig. 4a–e. In comparison to iron standards (Fe, FeO, Fe_2_O_3_, and Fe_3_O_4_), it could be seen that the iron valence in all *Bacillus* and *E. coli* cells belonged to Fe(III). These results suggested that Fe^0^ NPs did enter the cells and became oxidized into FeO, Fe_2_O_3_, and Fe_3_O_4_. The XANES spectra of two *Bacillus* and two *E. coli* strains overlapped with the spectra of Fe_2_O_3_ and Fe_3_O_4_ from 7100 to 7200 eV, particularly at the absorption peak of the iron species (7135 eV). To compare the oxidation states for the two strains and the Fe_2_O_3_ and Fe_3_O_4_ standards, the absorption peaks from 7125 to 7160 eV were enlarged, as shown in Fig. 4a–e. The absorption peaks of both strains at 7135 eV were similar to those of Fe_2_O_3_, indicating that iron had valence Fe(III) in both cases. However, it is difficult to compare the valence of iron species between the two *E. coli* strains (Fig. 4d, e).

**Fig. 5:**
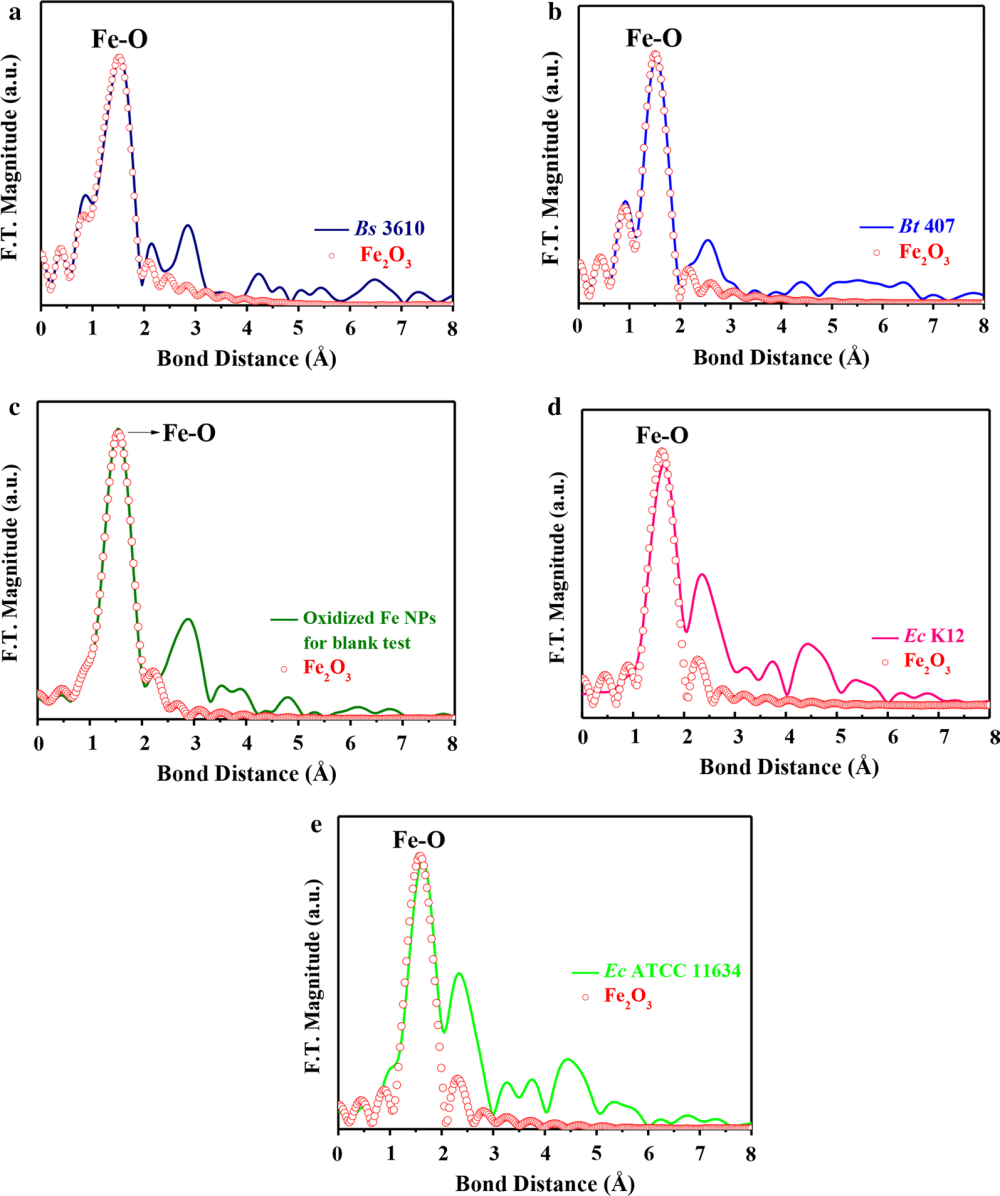
Fe *K*-edge Fourier-transformed (F.T.) EXAFS spectra of **a** *B. subtilis* 3610 (*Bs *3610). **b** *B. thuringiensis* 407 (*Bt *407). **c** Oxidized Fe(ZVI) NPs. **d** *E. coli* K12 (*Ec* K12). **e** *E. coli* ATCC11634 (*Ec* ATCC11634) fitted with the Fe_2_O_3_ structural model. Data are representative of two separate experiments

**Fig. 6:**
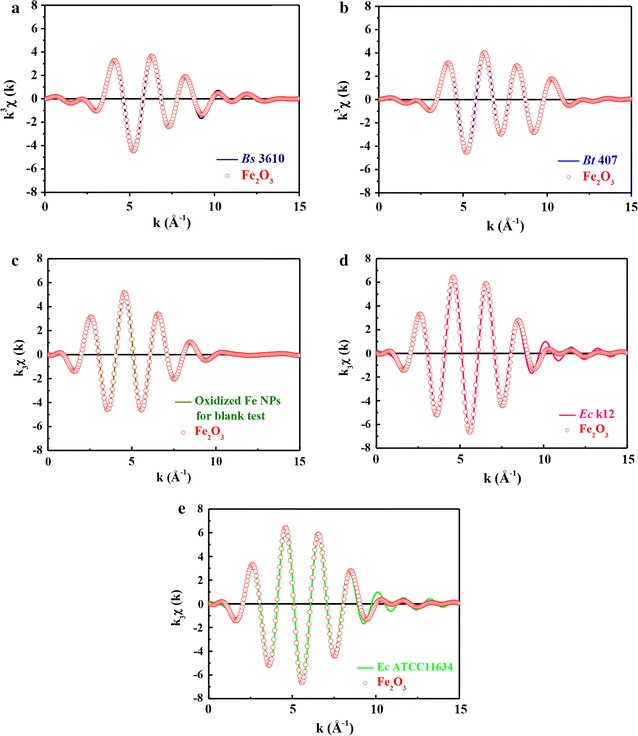
Fe *K*-edge EXAFS oscillation k^3^χ (k) of **a** *B. subtilis* 3610 (*Bs *3610), **b** *B. thuringiensis* 407 (*Bt *407), **c** oxidized Fe(ZVI) NPs, **d** *E. coli* K12 (*Ec* K12), and **e** *E. coli* ATCC11634 (*Ec* ATCC11634) fitted with the Fe_2_O_3_ structural model. Data are representative of two separate experiments

To further characterize and confirm Fe_2_O_3_ in the two *Bacillus* strains, two *E. coli* strains, and oxidized ZVI NPs, EXAFS spectra were obtained. As shown in Fig. 5a–e, the Fe–O bond distance of the first shell in both *Bacillus* strains was consistent with that of the Fe_2_O_3_ standard. Therefore, the atomic neighbors, bond distance, and coordination number of Fe_2_O_3_ in the two *Bacillus* strains, two *E. coli* strains, and oxidized ZVI NPs could be analyzed according to the fine structural parameters of Fe_2_O_3_. A similar overlap with the Fe_2_O_3_ standard was observed also for k^3^-weighted and least squares-fitted Fe_2_O_3_ K-edge fine structure inverse Fourier-transform oscillation spectra of both strains (Fig. 6a–e). Detailed structural parameters of Fe_2_O_3_ in two *Bacillus* strains, two *E. coli* strains, and oxidized ZVI NPs are given in Table 3. R-factor values were much smaller than 0.02, demonstrating the great reliability of EXAFS analysis. Generally, the coordination number (CN) of iron atoms is larger in oxides than that in metals. It is ascribed to the occupation of oxygen atoms near central iron atom in crystal structure. In particular, the coordination number for *B. subtilis* 3610 was larger than that for *B. thuringiensis* 407 and *E. coli* strains, indicating more neighboring oxygen atoms around the central Fe atoms in *B. subtilis* 3610. Accordingly, these extra oxygen atoms occupy the limited crystal structure space, which explains the shorter Fe–O bond distance than that detected in *B. thuringiensis* 407 and *E. coli* strains. Overall, the results of XANES and EXAFS analyses suggested that ZVI NPs were likely oxidized when in contact with the oxygen present inside bacteria, which then caused their death.

**Table 3:** Fine structural parameters of *B. subtilis* 3610, *B. thuringiensis* 407, oxidized Fe NPs, *E. coli* K12, and *E. coli* ATCC11634

Sample	Shell (1st)	CN (± 0.05 Å)^a^	R (± 0.01 Å)^b^	Δ σ^2^ (Å^2^)^c^	R factor
*Bs* 3610	Fe–O	5.63	1.97	0.01117	0.00427
*Bt* 407	Fe–O	3.74	1.99	0.00545	0.00327
Oxidized Fe(ZVI) NPs	Fe–O	4.23	1.98	0.01093	0.00027
*Ec* K12	Fe–O	3.67	1.98	0.00295	0.00226
*Ec* ATCC11634	Fe–O	3.66	1.99	0.00226	0.00544

^a^ *CN* coordination number

^b^ *R* bond distance

^c^ *σ* Debye–Waller factor

## Discussion

In this study, we found that 1000 ppm ZVI NPs was lethal to the two *Bacillus* strains but not to the two *E. coli* strains tested. At 1000 ppm, *B. thuringiensis* 407 was more resistant than *B. subtilis* 3610 to ZVI NPs, whereas no significant decrease was observed for either when treated with 500 ppm ZVI NPs. These results suggested that gram-negative strains may be more resistant to ZVI NPs than gram-positive strains.

Lee et al. [16] reported that 9 ppm ZVI NPs of 10–80 nm (average diameter ~ 35 nm) were toxic to gram-negative *E. coli* ATCC8739, resulting in 2.3-log inactivation per mg/L/h of cells grown in 2 mM carbonate buffer at pH 8.0 in the absence of oxygen. However, under aerobic conditions, 90 ppm ZVI NPs caused around 0.029-log inactivation per mg/L/h of cells. This finding suggested that ZVI NPs might exert significant antimicrobial effect only under anaerobic conditions. Notably, Lee et al. [16] used an atypical antimicrobial assay with an artificial carbonate buffer solution at a relatively more alkaline pH (pH 8), which was assessed under anaerobic conditions. This setting is not ideal for determining the practical minimum bactericidal concentration because *E. coli* does not grow very well in the absence of oxygen and at pH 8. In nature, *E. coli* cells live in aerobic environments with a pH close to 7. Thus, this method is likely too artificial to accurately measure cell death. Instead, in this study, we used the minimum bactericidal concentration method, a more common antimicrobial assay. Viability was determined by plating the cells and counting cell numbers. Even with 1000 ppm ZVI NPs at pH 7 and pH 8 (data not shown), the viability of both *E. coli* strains was comparable to that of untreated controls, whereas the viability of both *Bacillus* strains decreased significantly when treated with either 500 or 1000 ppm ZVI NPs at pH 7.

Diao and Yao [9] found that treatment with 100–1000 ppm of 20–30-nm ZVI NPs reduced *B. subtilis* viability by 80–100%, representing a 2-log reduction. In contrast, in the present study we found a 4-log reduction in viability with 1000 ppm ZVI NPs of a smaller size. This discrepancy suggested that size may influence the ZVI NP antimicrobial effect, with different species and strains displaying variations in the extent of resistance or susceptibility to ZVI NPs. Despite the clear difference in resistance between gram-positive and gram-negative strains reported here, gram-negative *P. stutzeri* is resistant to ZVI NPs, whereas *E. coli* and *P. fluorescens* are more sensitive [18], making it difficult to conclude that gram-negative strains are more sensitive to ZVI NPs.

To elucidate the mechanism through which ZVI NPs decrease bacterial cell growth, we stained *Bacillus* cells with the fluorescent dyes RedoxSensor and PI to test whether the nanoparticles decreased reductase activity and disrupted bacterial cell membranes. The observed concentration-dependent increase in membrane damage suggested that the nanoparticles increased membrane permeability and possibly penetrated cells to cause protein toxicity. Moreover, the observed decrease in reductase activity after exposure to ZVI NPs suggested that increased levels of ROS contributed to loss of membrane integrity and cell viability.

Therefore, the high concentrations of ROS generated by ZVI NPs may have the most deleterious effects on bacterial cells [36, 38–40]. ROS damage iron-sulfur groups in the cofactors of many enzymes, leading to the production of even more ROS via the Fenton reaction and resulting in cell injury and death. Importantly, we have now clearly demonstrated that ZVI NP-induced toxicity is related to an increase in oxidation and ROS. Upon internalization, ZVI NPs may be oxidized to Fe_2_O_3_, which could further promote ROS production to alter the redox status and kill the bacterial cells [41]. This process may explain the observed decrease in reductase activity and the increase in PI staining in both *Bacillus* strains.

XANES/EXAFS measurements showed that Fe_2_O_3_ was present in high amounts in bacterial cells incubated with ZVI NPs. In addition, all cells incubated with pre-oxidized ZVI NPs that became Fe_2_O_3_ were nontoxic to all strains, suggesting that ZVI NPs entered cells and were oxidized to Fe_2_O_3_, resulting in cell death, but were not oxidized outside cells. This was further confirmed with the PI and RedoxSensor assays. Overall, our results suggested a toxic mechanism through which ZVI NPs enter *Bacillus* cells and induce a ROS response, resulting in decreased RedoxSensor activity, with subsequent oxidation to Fe_2_O_3_.

However, *E. coli* strains were more resistant to ZVI NPs or the ROS response. On the basis of XPS, XANES, and EXAFS analyses, ZVI NPs were completely oxidized to FeO (Fe(II)) in MHB medium within a short time and then continuously oxidized, forming a relatively thick outer layer of Fe_2_O_3_ (Fe(III)). Moreover, the ZVI NPs were absorbed through monolayer cell membranes of *B. subtilis* 3610 and *B. thuringiensis* 407 and then became oxidized to FeO and Fe_2_O_3_ within the cells. Some ZVI NPs were accumulated inside of *B. subtilis* 3610 and *B. thuringiensis* 407 and could be easily detected by XPS. During the duration of ZVI NP oxidation, electrons were generated, exciting the chemical species inside bacteria into free radicals. Subsequently, *B. subtilis* 3610 and *B. thuringiensis* 407 were killed by these internal free radicals. Apart from the generation of free radicals, the consumption of oxygen within cells may be another reason for the death of *B. subtilis* 3610 and *B. thuringiensis* 407. For *E. coli* K12 and *E. coli* ATCC11634, ZVI NPs could not easily attach to the cell surface. Only a few ZVI NPs were oxidized near the surface of *E. coli* K12 and *E. coli* ATCC11634 and then coincidentally entered the cells via tiny cracks in the bilayer cell membrane.

This might explain the results observed in *Bacillus* cultures treated with ≥ 500 ppm ZVI NPs, whereby RedoxSensor activity was significantly decreased but membrane permeability was significantly increased. To the best of our knowledge, this is the first study to directly analyze ZVI NPs internalized in bacterial cells using XANES and EXAFS and to demonstrate that ZVI NP oxidation was the primary source of their toxicity [30, 31, 42]. ZVI NPs have been used for different applications in soil remediation [43–45]. The findings of this study provide new molecular insights into the effects of ZVI NP toxicity on gram-positive and gram-negative bacteria for application in ecotoxicological tests.

## Conclusion

To the best of our knowledge, this is the first study demonstrating the toxicity of ZVI NPs (at 1000 ppm) against gram-positive *B. subtilis* and *B. thuringiensis* but not gram-negative *E. coli* strains in the soil. In addition, XANES/EXAFS data showed that Fe_2_O_3_ was present in high amounts in the *Bacillus* cells cultured with ZVI NPs. Our findings suggested that ZVI NPs enter *Bacillus* cells and induce a ROS response, resulting in decreased RedoxSensor activity, with subsequent oxidation to Fe_2_O_3_. ZVI NPs have been used for different applications in soil remediation [43–45]; however, few studies have looked at the persisting challenges of such use and evaluated the biological interactions of NPs. The present findings may provide a molecular approach to elucidate the effects of ZVI NP toxicity on gram-positive and gram-negative bacteria for applications in ecotoxicological tests.

## Abbreviations

ZVI NPs: zero-valent iron nanoparticles
ROS: reactive oxygen species
MHB: Mueller–Hinton broth
FE-SEM: field-emission scanning electron microscopy
XRD: X-ray diffraction
PI: propidium iodide
XPS: X-ray photoelectron spectroscopy
XANES: X-ray adsorption near-edge spectra
EXAFS: extended X-ray adsorption fine structure
HR-TEM: high-resolution transmission electron microscopy
LB: Luria–Bertani
ANOVA: analysis of variance
CMA: cylindrical mirror analyzer
PBS: phosphate-buffered saline
